# Isolation, Phylogenetic and Gephyromycin Metabolites Characterization of New Exopolysaccharides-Bearing Antarctic Actinobacterium from Feces of Emperor Penguin

**DOI:** 10.3390/md19080458

**Published:** 2021-08-12

**Authors:** Hui-Min Gao, Peng-Fei Xie, Xiao-Ling Zhang, Qiao Yang

**Affiliations:** 1 College of Marine Science and Technology, Zhejiang Ocean University, Zhoushan 316022, China; gaohuimin@zjou.edu.cn (H.-M.G.); xiepengfei@zjou.edu.cn (P.-F.X.); 2 ABI Group, Zhejiang Ocean University, Zhoushan 316022, China; 3 Department of Environment Science and Engineering, Zhejiang Ocean University, Zhoushan 316022, China

**Keywords:** antarctic actinobacteria, gephyromycin, microbial bioflocculants, bacterial exopolysaccharides, gut microbiota, antarctic emperor penguin

## Abstract

A new versatile actinobacterium designated as strain NJES-13 was isolated from the feces of the Antarctic emperor penguin. This new isolate was found to produce two active gephyromycin analogues and bioflocculanting exopolysaccharides (EPS) metabolites. Phylogenetic analysis based on pairwise comparison of 16S rRNA gene sequences showed that strain NJES-13 was closely related to *Mobilicoccus pelagius* Aji5-31^T^ with a gene similarity of 95.9%, which was lower than the threshold value (98.65%) for novel species delineation. Additional phylogenomic calculations of the average nucleotide identity (ANI, 75.9–79.1%), average amino acid identity (AAI, 52.4–66.9%) and digital DNA–DNA hybridization (dDDH, 18.6–21.9%), along with the constructed phylogenomic tree based on the up-to-date bacterial core gene (UBCG) set from the bacterial genomes, unequivocally separated strain NJES-13 from its close relatives within the family Dermatophilaceae. Hence, it clearly indicated that strain NJES-13 represented a putative new actinobacterial species isolated from the gut microbiota of mammals inhabiting the Antarctic. The obtained complete genome of strain NJES-13 consisted of a circular 3.45 Mb chromosome with a DNA G+C content of 67.0 mol%. Furthering genome mining of strain NJES-13 showed the presence of five biosynthetic gene clusters (BGCs) including one type III PKS responsible for the biosynthesis of the core of gephyromycins, and a series of genes encoding for bacterial EPS biosynthesis. Thus, based on the combined phylogenetic and active metabolites characterization presented in this study, we confidently conclude that strain NJES-13 is a novel, fresh actinobacterial candidate to produce active gephyromycins and microbial bioflocculanting EPS, with potential pharmaceutical, environmental and biotechnological implications.

## 1. Introduction

Actinobacteria are a unique prokaryote group and a virtually unlimited source due to their extraordinary abilities to deliver a multitude of novel bioactive lead compounds with medical, pharmaceutical, industrial and ecological significance [1,2]. They are widespread in nature and have been recovered from a wide variety of terrestrial or aquatic habitats, as saprophytes, symbionts or pathogens [3]. Moreover, marine ecosystems are largely an untapped, promising source for deriving various kinds of rare actinobacteria which uniquely possess quite different biological properties, including antimicrobial, anticancer, antiviral, insecticidal and enzyme inhibitory activities [4]. Among them, the family Dermatophilaceae was first described by Van Saceghem in 1915 with *Dermatophilus* as the genus [5]. Recently, this family was reclassified into the newly proposed order *Dermatophilales* in the newly proposed class *Actinobacteria* [6]. So far, this family embraces seven genera which were isolated from the feces of mammalians [7], skins of infected mammalians [8], intestinal tracts of fishes [9], lake sediments [10] and also an activated sludge [11]. Interestingly, unusual novel bioactive Dermatophilaceae members with great potential for natural drug discovery were also isolated from marine sponges [12]. However, no bacterial strain belonging to this family has been reported from the Antarctic habitat yet. 

Bacteria produce a wide range of varied exopolysaccharides (EPS) which comprise a substantial component of the extracellular polymers surrounding most microbial cells, and function as a strategy for bacterial growth, adherence and survival under adverse stress conditions [13,14]. Marine EPS-bearing microbes inhabiting extreme niches have been found in cold environments, typically of Arctic and Antarctic sea ices [15], and hypersaline habitats such as salt lakes and salterns [16,17]. Indeed, microbial EPS derived from the extremophiles have shown diverse biotechnological promise, ranging from pharmaceutical industries for their immuno-modulatory and antiviral effects, and bone regeneration and cicatrizing capacity, to food industries for their peculiar gelling and thickening properties [13,18,19]. Moreover, some EPS are employed as novel microbial biosurfactants which can outcompete the traditional chemical flocculants due to their non-toxicity and extraordinary biodegradability characteristics [20,21,22]. 

Gephyromycin, the first intramolecular ether-bridged angucyclinone, was originally isolated from an antarctic *Streptomyces* strain named NTK-14 in 2005 [23]. Gephyromycin and its analogues exhibit powerful glutaminergic activity towards neuronal cells with a comparable effective dosage against DCG-IV, which is the most potent glutamate agonist [24]. It also has an obvious anti-tumor effect towards PC3 cells as a novel inhibitor of HSP90 [25]. However, gephyromycin has not been reported in all Dermatophilaceae members yet. In this study, we present the isolation of a novel versatile actinobacterium designated strain NJES-13 from the feces of antarctic emperor penguin. The new isolate produces gephyromycin analogues along with active bioflocculanting EPS metabolites. Based on our phylogenetic characterizations, strain NJES-13 represents a putative new actinobacterial species within the family Dermatophilaceae. Additionally, it is the first report for the bacterial species within this family isolated from the Antarctic habitat. Additionally, whole-genome sequencing and genome mining of the biosynthesis gene clusters (BGCs) of strain NJES-13 give insight into the practical application of this novel versatile antarctic actinobacterium for the production of the promising and active gephyromycin metabolites and EPS with potential pharmaceutical, environmental and biotechnological implications.

## 2. Results and Discussion

### 2.1. Phenotypic Characteristics of Strain NJES-13 

**Figure 1:**
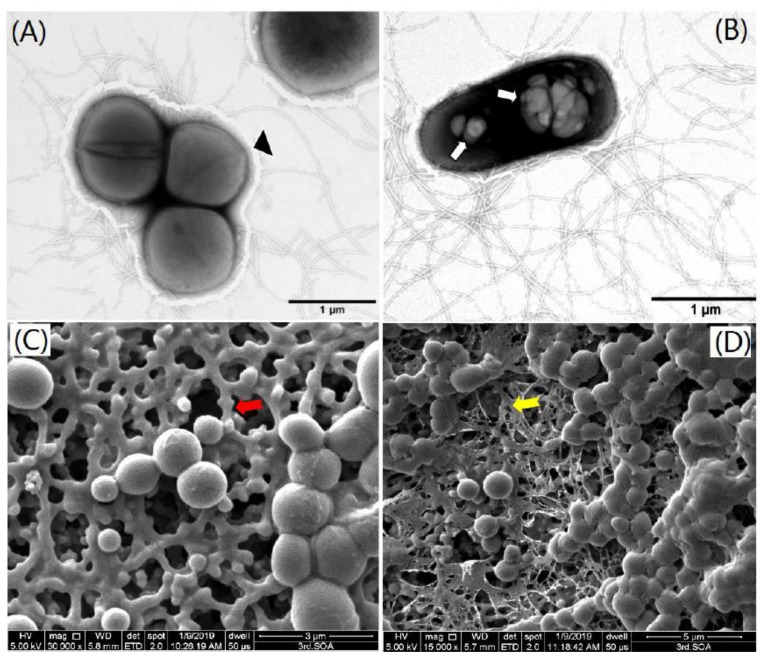
Morphological images of strain NJES-13 grown on marine R_2_A plates cultured at 28 °C for 2 days, bacterial morphology obtained transmission electron microscopy observation showing the coccoid-shaped cells with clusters with black arrows indicating the exopolysaccharides (EPS) layer (**A**), and (**B**) short rod-shaped cells showing the polyhydroxyalkanoates (PHA) granules (white arrows) inside the cells observed by scanning electron microscopy (SEM) showing the coccoid-shaped cells with clusters interconnected by thicker (**C**) and thin (**D**) viscous EPS showing three-dimensional net-like morphology (red/yellow arrows). Bar, 1 μm for pane A and B, 3 μm for pane C and 5 μm for pane D.

Cells of strain NJES-13 were observed to form yellow colonies when grown on marine R_2_A plates at 28 °C for 2 days. Cells were Gram-negative, non-sporulating and motile with the peritrichous flagella (Figure 1A,B) with a faint layer of extracellular slime around the cells (Figure 1A), aerobic and weakly positive for anaerobic growth. This strain developed clusters of coccoid approximately 0.6–1.1 μm in diameter. It can divide by binary fission (Figure 1C and Figure S1) in the early phase of growth, and then the cell clusters were gradually disrupted during the stationary phase to form short rod-shape cells containing polyhydroxyalkanoates (PHA) granules inside (Figure 1B). The rod-shaped cells were approximately 0.5–0.8 μm wide and 0.7–1.2 μm long, and with clusters interconnected by the thicker (Figure 1C), or thin (Figure 1D) viscous EPS showing a three-dimensional net-like morphology. Bacterial growth of strain NJES-13 was observed to occur at 15–45 °C at pH 6.0–9.0 in the presence of 0.5–9% (*w/v*) NaCl. 

### 2.2. Gephyromycin Metabolites Characterization

**Figure 2:**
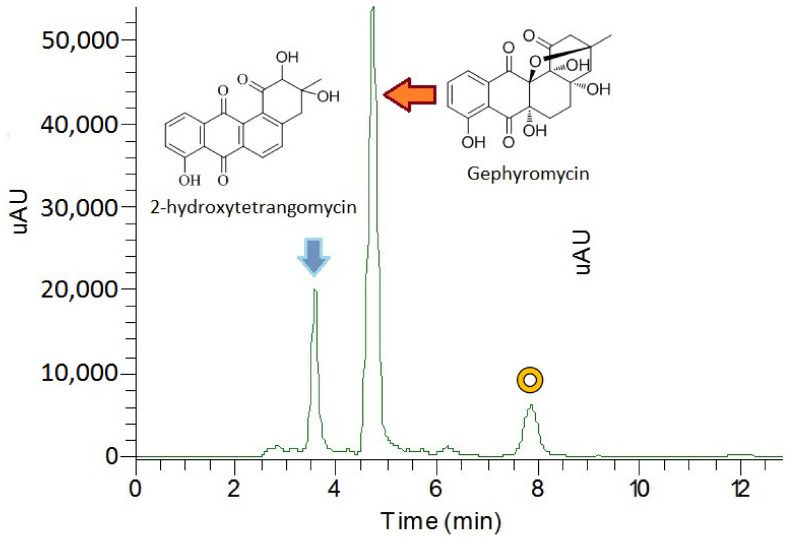
HPLC profile of the metabolites of strain NJES-13 after 10 days culture in R_2_A medium at 28 °C. About 250 mg of wet cultures at exponential phase was extracted and then analyzed by RP-HPLC (C_18_, 5 μm, 10 mm × 250 mm, I.D.) using a gradient elution ranging from 0 to 100% MeOH/H_2_O in 20 min, and recorded at 290 nm. The peak marked with a brown circle was a potential new gephyromycin analogue whose chemical structure remains to be elucidated.

Two compounds were isolated from the culture of strain NJES-13. The extracts were separated by HPLC (Figure 2) and identified based on the data obtained by combined characterization based on high-resolution electrospray ionization mass spectroscopy (HR-ESI-MS) and ^1^H and ^13^C NMR analysis (Table S1). Compound 1 was identified as the known 2-hydroxytetrangomycin by HR-ESI-MS analysis to obtain a molecular formula, C_19_H_15_O_6_ (m/z 339.0881 [M+H]^+^, calculated value 339.0863). Additionally, compound 2 was identified as gephyromycin with the molecular formula, C_19_H_19_O_8_ (*m/z* 375.1096 [M+H]^+^, calculated value 375.1074). The obtained ^1^D ^1^H and ^13^C NMR data were consistent with those reported in the literature for the two known compounds [24,25]. Moreover, the extra peak (retention time at 7.95 min) that appeared in the HPLC chromatogram was a potential new gephyromycin analogue (data not shown), but its detailed chemical structure remains to be elucidated due to its low production level cultured in the unoptimized R_2_A media. According to the quantitative analysis, the production level of 2-hydroxytetrangomycin and gephyromycin in strain NJES-13 were calculated as 3.056 and 2.554 mg·L^−^^1^, respectively. The bioactivities of gephyromycin analogues have been demonstrated in previous reports [23,24,25]. This finding indicates the new isolate may provide a new antarctic actinobacterium candidate for the production of those promising active gephyromycins.

### 2.3. Whole-Genome Sequencing and Annotation

**Figure 3:**
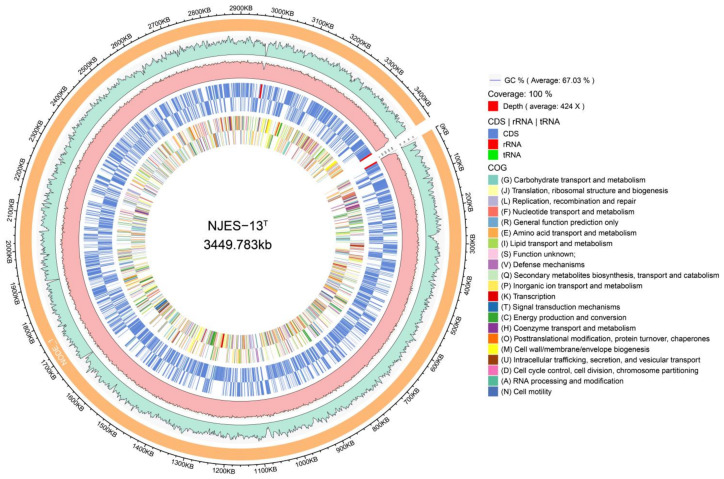
Circular representation of the single chromosome of strain NJES-13 genome using Circos tool (version 0.64, BCC, Vancouver, BC, Canada). The scale of the genome size is shown in the outer line. From the outer to inner circle: the two outer circles show the predicted protein-coding sequences (CDs) on the plus and minus strand. Different colors in these two circles show genes with different COG categories; the third circle shows rRNA and tRNA (red); and the fourth and inner fifth circles show G + C content and G + C skew, respectively.

The genomic size of strain NJES-13 was 3,449,783 bp with a calculated G+C content of 67.0%, an *N*_50_ value of 3,449,783 bp and an over 420× genome coverage. A circular representation of its complete genome, including one chromosome, is shown in Figure 3. Based on the genome annotation, the total number of predicted genes was 3082, of which 3026 were protein-coding genes. It also had 56 RNA genes including 47 tRNA and 9 rRNA genes. A total of 79 CRISPR sequences were found in the chromosome. Then, 2197 genes (74.5%) were assigned to 26 functional categories of the COG database, and 850 genes participated in the pathway of metabolism which was the primary function of the chromosome. Additionally, total 143 genes were not assigned any known function, accounting for about 6.5% of the total. KEGG analysis revealed 1550 proteins involved in five pathways, in which about 42.8% were classified into the “metabolism” category. GO analysis assigned 2177 proteins into three functional terms in which the top five subgroups were plasma membrane, integral to membrane, ATP binding, cytoplasm and metal ion binding, respectively (Table S2).

### 2.4. Phylogenetic Analysis

**Figure 4:**
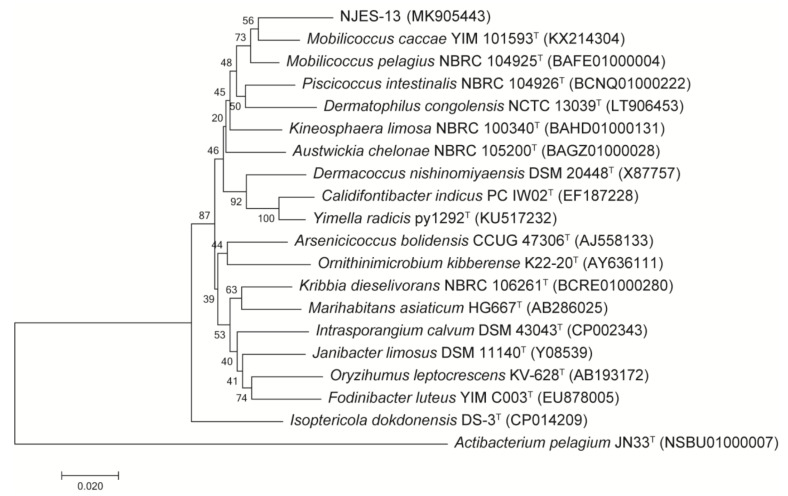
Phylogenetic tree constructed using neighbor-joining (NJ) method based on the 16S rRNA gene sequences of strain NJES-13 and its close relative type strains within the family Dermacoccaceae. Bootstrap values are expressed as percentages of 1000 replicates. *Actibacterium pelagium* JN33^T^ was used as the outgroup. Bar, 0.02 substitutions per nucleotide position.

Phylogenetic analysis based on the bacterial 16S rRNA gene sequences between strain NJES-13 and its close relatives were performed. The gene similarity identification based on the 16S gene alignment indicated that strain NJES-13 showed the highest gene sequence similarity with *Mobilicoccus pelagius* Aji5-31^T^ (95.9%), which was obviously far lower than the threshold (98.65%) generally recognized for novel species delineation [26,27]. Moreover, phylogenetic trees using the neighbor-joining (NJ) method demonstrated that strain NJES-13 was closely related to the *Mobilicoccus* group compared with all other type strains within the family Dermatophilaceae (Figure 4). 

### 2.5. Phylogenomic Calculations

**Figure 5:**
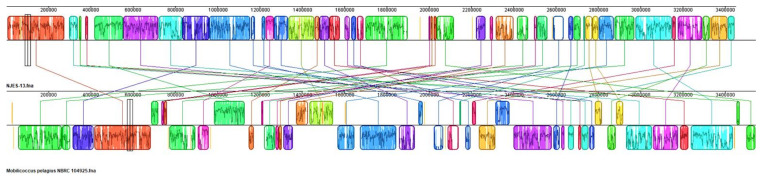
Genome organizations of strain NJES-13 and the type strain of *Mobilicoccus pelagius.* Each genome is laid out horizontally with homologous segments (LCBs) outlined as colored rectangles. Lines with the same color collate aligned segments between the two genomes. Average sequence similarities within an LCB, measured in sliding windows, are proportional to the heights of interior colored bars. Large sections of white within blocks and gaps between blocks indicate lineage-specific sequence.

**Figure 6:**
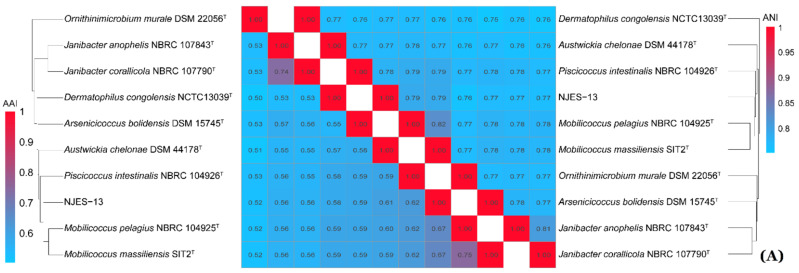

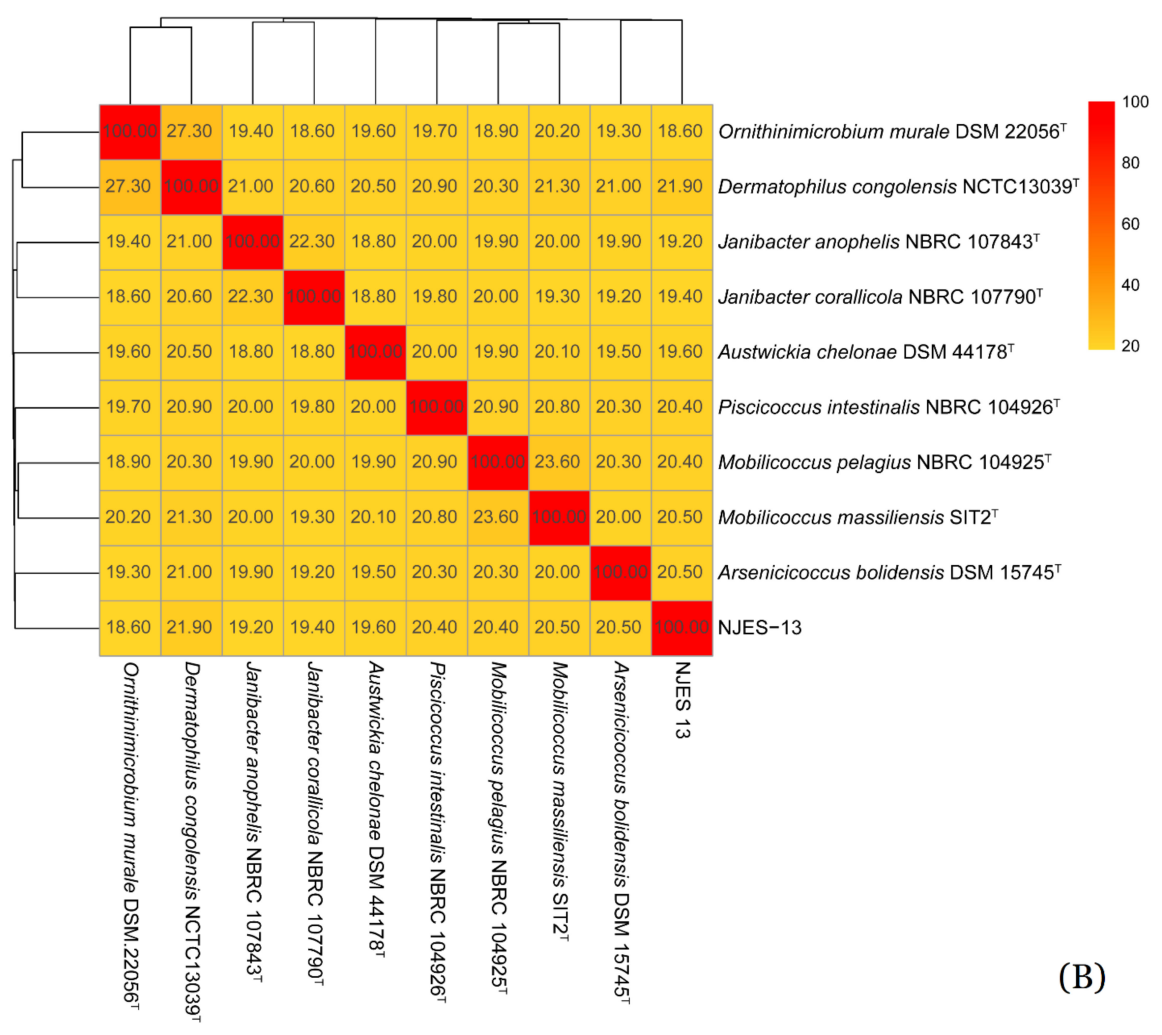
Phylogenomic calculations of the ANI/AAI (**A**) and dDDH (**B**) values among the type species with available genomes within the family Dermatophilaceae.

**Figure 7:**
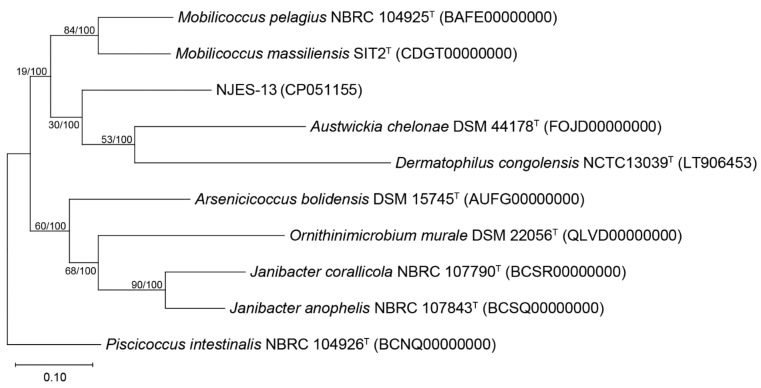
Phylogenomic tree of strain NJES-13 and its close relatives based on the up-to-date bacterial core gene (UBCG) set. Bar, 0.10 nucleotide substitutions per site.

In order to identify the maximal matches and local collinear blocks (LCBs), a multiple whole-genome alignment of genome sequences of strains NJES-13 and its closest relative *Mobilicoccus pelagius* Aji5-31^T^ was performed using PATRIC software [28]. As shown in Figure 5, the LCBs alignment identified 44 conserved gene regions larger than 1 kb, which demonstrated clear differences from each other. Moreover, for completely assuring the taxonomic position of the new isolate, the phylogenomic comparisons based on average nucleotide identity (ANI), average amino acid identity (AAI) and digital DNA–DNA hybridization (dDDH) values between strain NJES-13 and other type strains within the family Dermatophilaceae with available genomes were performed. The obtained calculation results are shown in Figure 6. The ANI and AAI values between NJES-13 and other type members of Dermatophilaceae with available genome sequences ranged from 75.9 to 79.1%, and 52.4 to 66.9%, respectively (Figure 6A), which are both below the thresholds proposed for different genera [28]. The dDDH (18.6–21.9%) values (Figure 6B) calculated between members of separate genera of this family, were also far below the threshold value for genera delineation [28]. These low phylogenomic values, along with 16S rRNA gene identity, support novel species delineation of strain NJES-13 from the close relatives. Additionally, based on the constructed phylogenomic UBCG tree (Figure 7), strain NJES-13 formed a separate branch clustered with the type species of the genera of *Austwickia* and *Dermatophilus,* and apart from the two *Mobilicoccus* species, although they shared the same root on the phylogenomic tree. It further confirmed the phylogenetic position as a putative new member within the family Dermatophilaceae (the detailed polyphasic taxonomic identification will be published elsewhere). Additionally, it’s the first report of the new actinobacterial species within the family Dermatophilaceae isolated from the gut microbiota of mammalians living in the Antarctic habitat.

### 2.6. Bioflocculanting Potential of Bacterial EPS

**Figure 8:**
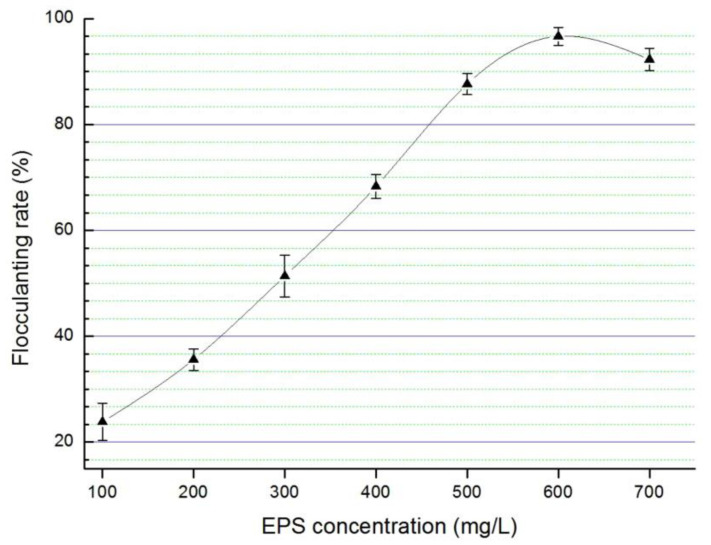
Concentration-dependent manner of bioflocculanting rates of the EPS produced by strain NJES-13.

Initially, the transmission electron microscope observation of strain NJES-13 cells clearly demonstrated the presence of an EPS layer outside of the cells (Figure 1). Then, the extracted EPS produced by strain NJES-13 were subjected to the bioflocculanting activity screening assay to evaluate their production capacity of bacterial bioflocculants [29,30,31]. The comparison of bioflocculanting effects under a series of EPS concentrations was performed. Figure 8 shows the concentration-dependent manner of bioflocculanting bioactivity of the EPS produced by strain NJES-13. It can be clearly seen that the bioflocculanting activity reached the maximum of 96.7 ± 1.8% (means ± SD) at the EPS concentration of 0.60 g·L^−1^. It exhibited a higher bioflocculanting capacity compared with other marine bacterial strains isolated from marine phycosphere niches that we previously reported [29,30,31]. These findings obviously indicate that strain NJES-13 may serve as a novel actinobacterial candidate with natural potential for the production of promising microbial bioflocculants derived from the unique mammalian gut microbiota niche in the antarctic habitat. Additionally, the ongoing chemical elucidation of the bioflocculanting EPS complex is believed to eventually reveal the chemical nature for the extraordinary natural instincts of strain NJES-13.

### 2.7. Biosynthetic Genes Responsible for Active Metabolites

Based on the whole-genome sequence of strain NJES-13, the antiSMASH tool was used to predict the biosynthetic gene clusters (BGCs) responsible for secondary metabolite synthesis [32]. According to the obtained prediction result, total five BGCs were found in the genome of strain NJES-13, including a 44.5 kb long type III polyketide synthase (T3PKS) cluster with 100% similarity, as well as one 47.4 kb long type I polyketide synthase (T1PKS) cluster, two NRPS-like clusters of 43.4 and 42.0 kb, respectively, and one 27.8 kb long beta-lactone cluster. These findings indicate that strain NJES-13 possesses great potential to produce novel bioactive metabolites [33,34,35,36]. But unlike its closest relative, both T3PKS and T1PKS gene clusters were absent in the type strain of *Mobilicoccus pelagius*.

For bacterial EPS biosynthesis, several biosynthesis genes (*wzx, exo* and *muc*) were found in the genome of strain NJES-13. However, they were also not found in the type strain of *Mobilicoccus pelagius*. Based on the bioflocculanting activity assay of EPS of strain NJES-13, it is indicated that strain NJES-13 dwelling in the gut microenvironment of antarctic emperor penguin might act as a novel fresh candidate with great production potential for natural promising bioflocculants [21,22,23]. Additionally, ultrastructure observation of the cells of strain NJES-13 by transmission electron microscopy also clearly showed the presence of polyhydroxyalkanoates (PHA) granules inside the cells (Figure 1). Furthermore, genomic mining of strain NJES-13 revealed one loci harboring genes assigned to PHA biosynthesis pathway. Bacterial PHAs are used to store carbon and energy, and highlight the huge talent for the production of biodegradable bioplastics [37,38]. These encoding genes included acetoacetyl-CoA reductase, poly(R)- hydroxyalcanoic acid synthase and an ORF-encoding phasin protein responsible for PHA granule formation. 

## 3. Materials and Methods

### 3.1. Bacterial Isolation and Culture

The feces of an Antarctic adult Emperor penguin were obtained on the terrestrial spot (69°22’24.78”S, 76°22’14.24”E) on December 10 in 2012 during the 29th Chinese National Antarctic Research Expedition (CHINARE), and sampled using sterile wooden spatulas and placed in a sterile tube. The obtained samples were brought back to the Biological Lab built in the Xuelong Expedition Ship [39]. For bacterial isolation, the feces samples were washed with sterile phosphate-buffered saline (PBS) buffer (pH 7.2). Cells were collected by centrifugation at 5000× *g* for 10 min, and re-suspended in 1 mL of sterile PBS buffer (pH 7.20). Aliquots of the cell suspension were then subjected to ten-fold serial dilution and plated in triplicate on marine R_2_A agar (Difco), and cultivated for 5–14 days at 28 °C. The isolated NJES-13 was routinely cultivated on the same medium in slant tubes at 28 °C and maintained as a glycerol suspension (30%, *v/v*), stored at −80 °C for long-term preservation. 

### 3.2. Phylogenetic Analysis of Bacterial 16S rRNA Genes

For 16S rRNA gene sequencing and phylogenetic analysis, the genomic DNA of the strain was extracted using a genomic DNA extraction kit (Promega; Shanghai, China). The universal bacterial primer pair of 27F/1492R was used for amplification of the 16S rRNA gene. PCR amplification was carried out according to the method described previously [40]. The PCR product was purified and cloned into pMD18-T vector (TaKaRa, Beijing, China) according to the manufacturer’s instructions. Plasmids were sequenced using universal primers (M13 forward and reverse) by MajorBio (Shanghai, China). The identification of phylogenetic neighbor and the calculation of 16S rRNA gene similarity were achieved using the EzTaxon server (http://www.ezbiocloud.net, accessed on 16 May 2021). Reference sequences of type strains were downloaded from the NCBI database. The 16S gene sequences of the type strains that were closely related to strain NJES-13 downloaded from the NCBI database were subjected to phylogenetic analysis using MEGA version 7.0 after multiple alignment of the data via ClustalW [41,42]. Phylogenetic distances were calculated according to neighbor-joining method. In each case, bootstrap values were calculated based on 1000 replications to evaluate the phylogenetic tree topology.

### 3.3. Bacterial Phenotypic Profile

Cell morphology of an exponentially growing culture of strain NJES-13 was observed by transmission and scanning electron microscopy (JEOL, Tokyo, Japan) [40]. The morphology and the colonies were observed on R_2_A after 24 h of incubation at 28 °C. Growth range and optimum at different temperatures (5–50 °C with 5 unit intervals) and pH values (4.0–11.0, at 0.5 unit intervals) was investigated in R_2_A media and cultured at 28°C for 2 days. Growth at various NaCl concentrations (0–11.0%, *w/v*, with interval of 0.5%) at pH 7.2 was investigated in R_2_A liquid medium at 28 °C for 2 days. Growth under anaerobic conditions was determined after incubation in an anaerobic chamber (Bactron EZ-2; Shellab) for 1 week on marine R_2_A plates cultivated at 28 °C. Oxidase and catalase activities were determined according to Yang et al. [42]. 

### 3.4. Characterization of Secondary Metabolites

Strain NJES-13 was cultured in 500 mL marine R_2_A liquid medium in 2 L glass flasks shaken at 150 rpm at 28 °C for 7 days. A total 2 L volume of culture broth was centrifuged at 6000 g. The harvested cells were frozen at −80 °C for 3 h, and then were extracted with 2 L cold pure acetone. The extraction procedure was repeated twice. The combined 4 L extracts were concentrated to about 200 mL volume under reduced pressure, and then subjected to the MCI column and eluted with gradient MeOH/H_2_O (10:90, 50:50, 80:20 and 100) to obtain three elution fractions. The 80% MeOH fraction was separated by Sephadex LH-20 gel column, and then eluted with ethanol to obtain two fractions, named fraction D (~26.5 mg) and E (~18.3 mg). The purification procedure was performed on an RP-HPLC system (C_18_, 5 μm, 10 mm × 250 mm, I.D.) applying a gradient elution ranging from 0 to 100% MeOH/H_2_O for fraction D to obtain compound 1 (~6.1 mg), and for fraction E to obtain compound 2 (~5.1 mg).

### 3.5. Whole-Genome Sequencing, Assembly and Annotation

Strain NJES-13 was cultured and maintained in marine R_2_A media (Difco) and cultured at 28 °C, and was harvested in the mid-logarithmic phase. The genome DNA was extracted and purified using the QIAamp DNA Mini Kit (Qiagen, Hilden, Germany) according to the manufacturer’s instruction, and sequenced by PacBio RS II Single Molecule Real-Time (SMRT) sequencing platform (Pacific Biosciences, Menlo Park, CA, USA) as described previously [42]. For PacBio sequencing, genomic DNA was sheared to 10 kb using g-TUBE (Covaris, Woburn, MA, USA) and converted into the proprietary SMRTbell^TM^ library format using PacBio RS DNA Template Preparation Kit. The library was sequenced on the SMRT platform. Total 1078 Mb filtered high-quality reads with average 424-fold genome coverage were assembled into one contig. The contig was connected into 1 chromosome by SOAPdenovo [43]. Based on the fine genome of strain NJES-13, the protein-coding regions were predicted by Glimmer (Version 3.02) [44], and all the gene functions were annotated by aligning to GO (http://www.geeontology.org, accessed on 28 April 2021), COG (https://www.ncbi. nlm.nih.gov/COG/, accessed on 30 April 2021) [45] and KEGG (http://www.kegg.jp, accessed on 30 April 2021) databases [46], respectively. Circular representation of the single chromosome of strain NJES-13 genome was performed using Circos (version 0.64, BCC, Vancouver, BC, Canada) (http://circos.ca, accessed on 12 April 2021) [47].

### 3.6. Phylogenomic Analysis 

The genome sequences of selected type strains within the family Dermatophilaceae were available until June 2021, and downloaded from GenBank genome database (https://www.ncbi.nlm.nih.gov/genome, accessed on 10 June 2021). The up-to-date bacterial core gene set (UBCG) was used to construct a phylogenetic tree using the genomes of strain NJES-13 and other reference type strains [21,48]. DNA G+C content of strain NJES-13 was calculated based on the genome sequence. Three measures of similarity based on the average nucleotide identity (ANI), average amino acid identity (AAI) and digital DNA–DNA hybridization (dDDH) values were calculated using online OrthoANI tool and GGDC tool (http://ggdc.dsmz.de/distcalc2.php, accessed on 12 June 2021), respectively, using the default parameters. A multiple whole-genome alignment of genome sequences to identify the maximal matches and local collinear blocks (LCBs) were performed using PATRIC software (https://www.patricbrc.org, accessed on 15 June 2021) [27].

### 3.7. Biosynthetic Gene Clusters Prediction

The in silico prediction of biosynthetic gene clusters (BGCs) for secondary metabolite synthesis was performed using Secondary Metabolite Analysis Shell (antiSMASH) version 6.0.0 [32] based on the whole-genome sequence of strain NJES-13.

### 3.8. Bacterial EPS Bioflocculanting Activity Assay

Extraction of bacterial EPS produced by strain NJES-13 and bioflocculanting activity evaluation were performed according to our procedures reported previously [20,49]. The prepared EPS were dissolved in distilled water for further bioflocculanting activity assay [23]. Briefly, the measurements using the kaolin clay suspension flocculation assay calculated as flocculation rate were used and performed in a 96-well microplate with a modified high-throughput manner in at least triplicate [20,21,22]. All the results are expressed as the means ± SD. The statistical significance was analyzed using *t*-test in SPSS Statistics (version 17.0, IBM, Shanghai, China) and plotted with Origin (verstion 8.0, Electronic Arts Inc., Redwood City, CA, USA). A value of *p* < 0.05 was considered statistically significant for all analyses [50].

### 3.9. GenBank Accession Numbers

The DDBJ/EMBL/GenBank accession number for 16S rRNA gene sequences of the strain NJES-13 is MH197128. The complete genome sequence of strain NJES-13 was deposited at DDBJ/EMBL/GenBank with the accession number CP051155.

## 4. Conclusions

Based on the phylogenetic analysis using the bacterial 16S rRNA gene alignment, strain NJES-13, isolated from the feces of Antarctic emperor penguin, was shown to belong to the family Dermatophilaceae. Additional phylogenomic evidence obtained unequivocally separated strain NJES-13 from its close relatives, and further confirmed the phylogenetic position of this isolate to represent a putative new actinobacterial species within the family Dermatophilaceae. It produces two active gephyromycin analogues and bioflocculanting exopolysaccharide metabolites. Accordingly, furthering genome mining showed the presence of biosynthetic gene clusters for those active bacterial metabolites. Thus, the combined phylogenetic and active metabolites characterization convincedly reveals that strain NJES-13 represents a novel, fresh actinobacterial candidate to generate natural microbial products with great potential pharmaceutical, environmental and biotechnological utilizations.

## Data Availability

The article contains all the data produced in this study.

## Supplementary Materials

The following are available online at https://www.mdpi.com/article/10.3390/md19080458/s1, Figure S1: Transmission electron microscopy (TEM) observation of the dividing cells of strain NJES-13 by binary fission with black arrows indicating the boundary of division. *Bar*, *1* μm. Table S1: The ^1^H (400 MHz, CDCl_3_) and ^13^C NMR (151 MHz, CDCl_3_) data for compounds 1 and 2. Table S2: Gene numbers of the functional categories of COGs based on the genomic sequence of strain NJES-13.
