# Supplementary material for: Isolation, Phylogenetic and Gephyromycin Metabolites Characterization of New Exopolysaccharides-Bearing Antarctic Actinobacterium from Feces of Emperor Penguin
Source: Mar Drugs. 2021 Aug 12;19(8):458. doi: 10.3390/md19080458 (PMC8400323; doi:10.3390/md19080458)
Supplement: Supplementary file 1 [file marinedrugs-19-00458-s001.zip › marinedrugs-1281830-supplementary.pdf]

Supplementary Materials

Isolation, Phylogenetic and Gephyromycin Metabolites Characterization of New Exopolysaccharides-bearing Antarctic Actinobacterium from Feces of Emperor Penguin

Hui-Min Gao [1], Peng-Fei Xie [1,2], Xiao-Ling Zhang [1,2,*] and Qiao Yang [1,2,3] *

[1] College of Marine Science and Technology, Zhejiang Ocean University, Zhoushan 316022, China; gaohuimin@zjou.edu.cn (H.-M.G.); xiepengfei@zjou.edu.cn (P.-F.X.);

[2] ABI Group, Zhejiang Ocean University, Zhoushan 316022, China

[3] Department of Environment Science and Engineering, Zhejiang Ocean University, Zhoushan 316022, China

*** Corresponding authors.**
E-mail addresses: zhangxiaoling@zjou.edu.cn (X.L.Z.); qiaoyang1979@whu.edu.cn (Q.Y.)

Figure S1 Transmission electron microscopy (TEM) observation of the dividing cells of strain NJES-13 by binary fission with black arrows indicating the boundary of division. *Bar, 1* μm

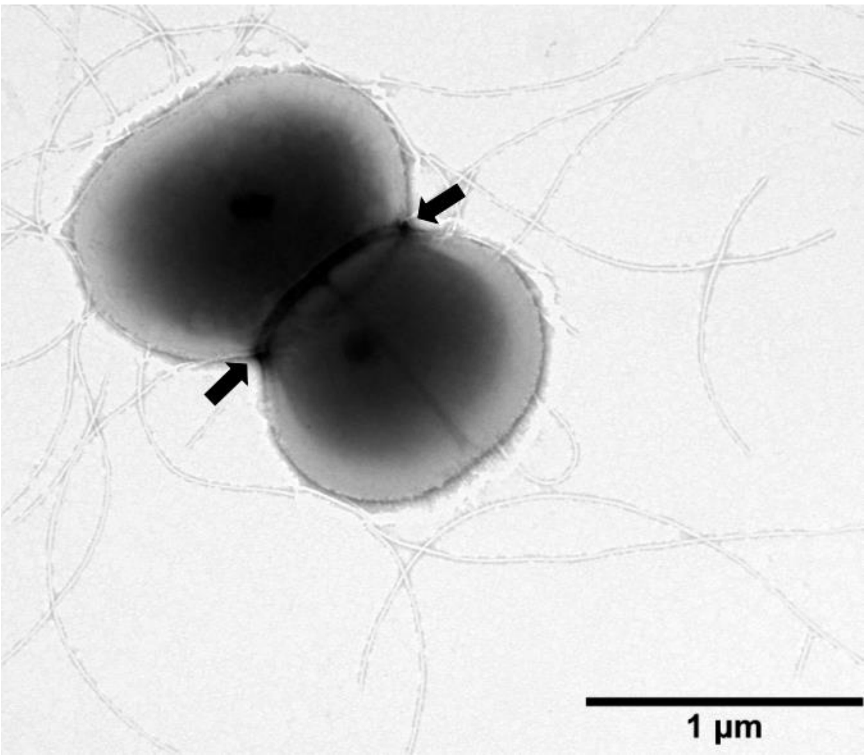

Table S1 The ^{1}H (400 MHz, $CDCl_3$) and ^{13}C NMR (151 MHz, $CDCl_3$) data for compounds 1 and 2

Compounds 1 (2-ydroxytetrangomycin)		Compounds 2 (gephyromycin)	
^{1}H	^{13}C	^{1}H	^{13}C
13.65-12.98 (m, 1H),	189.14,	11.45 (s, 1H),	204.10,
12.86-12.41 (m, 1H),	161.33,	7.62 (d, *J* = 8.0 Hz, 1H),	197.84,
8.11-7.76 (m, 1H),	140.13,	7.58 (d, *J* = 1.3 Hz, 1H),	191.73,
7.74-7.62 (m, 1H),	136.74,	7.30 (dd, *J* = 8.2, 1.3 Hz, 1H),	161.61,
7.26 (s, 2H),	126.09,	4.74-4.47 (m, 2H),	136.01,
5.73-4.93 (m, 1H),	118.80,	2.58 (d, *J* = 6.7 Hz, 2H),	132.41,
3.31-2.91 (m, 2H),	115.94,	2.34 (d, *J* = 2.5 Hz, 3H),	124.26,
2.87-2.42 (m, 2H),	77.16,	2.15-2.02 (m, 1H),	118.83,
2.50-2.16 (m, 2H),	76.95,	1.97 (d, *J* = 14.7 Hz, 1H),	115.05,
2.14-1.81 (m, 1H),	76.81,	1.89-1.78 (m, 2H),	79.67,
1.56-1.10 (m, 11H),	72.16,	1.23 (s, 3H)	70.82,
1.10-0.61 (m, 4H)	36.94,		46.35,
	28.84,		29.28,
	22.67		25.02

Table S2 Genes numbers of the functional categories of COGs based on the genomic sequence of strain NJES-13

	Functional group	Gene number	Gene ratio,%
A	RNA processing and modification	1	0.05
C	Energy production and conversion	147	6.69
D	Cell cycle control, cell division, chromosome partitioning	19	0.86
E	Amino acid transport and metabolism	205	9.33
F	Nucleotide transport and metabolism	66	3.00
G	Carbohydrate transport and metabolism	157	7.15
H	Coenzyme transport and metabolism	122	5.55
I	Lipid transport and metabolism	102	4.64
J	Translation, ribosomal structure and biogenesis	152	6.92
K	Transcription	148	6.74
L	Replication, recombination and repair	110	5.01
M	Cell wall/membrane/envelope biogenesis	125	5.69
N	Cell motility	15	0.68
O	Posttranslational modification, protein turnover, chaperones	72	3.28
P	Inorganic ion transport and metabolism	149	6.78
Q	Secondary metabolites biosynthesis, transport and catabolism	49	2.23
R	General function prediction only	261	11.88
S	Function unknown	143	6.51
T	Signal transduction mechanisms	80	3.64
U	Intracellular trafficking, secretion, and vesicular transport	27	1.23
V	Defense mechanisms	47	2.14
